# A Survey on the Actual Use of and Reasons for Heated Tobacco Products in Patients with Rheumatoid Arthritis

**DOI:** 10.3390/ijerph191912465

**Published:** 2022-09-30

**Authors:** Hisaaki Isaji, Kiyofumi Yamada

**Affiliations:** Department of Neuropsychopharmacology and Hospital Pharmacy, Nagoya University Graduate School of Medicine, Nagoya 466-8560, Japan

**Keywords:** heated tobacco product, rheumatoid arthritis, psoriasis, chronic inflammatory diseases, internet survey, chronic obstructive pulmonary disease, reason for use

## Abstract

The actual use of heated tobacco products (HTPs) among smokers with rheumatoid arthritis (RA) is little known. The objective of this study was to clarify the prevalence and reasons for HTP use among smokers with RA. We administered a web survey to a research company panel in Japan between December 2020 and January 2021. After 170,000 panelists completed a primary questionnaire regarding smoking and disease status, 198 smokers with RA completed a secondary questionnaire regarding the reasons for HTP use and perceptions about the harmfulness of HTPs. The primary questionnaire revealed that smokers with RA were more likely to use HTPs than smokers without RA, and the adjusted odds ratio of RA for HTP use was one of the highest factors among other diseases (adjusted OR: 2.34, 95% CI: 1.92–2.85, *p* < 0.001). The secondary questionnaire revealed that 43.7% of HTP smokers with RA had considered using HTPs due to their RA, and 42.0% of them felt that starting HTPs relieved the symptoms of RA. These results indicated that smokers with RA tend to start using HTPs due to their RA, despite the lack of evidences that HTPs are safer alternatives.

## 1. Introduction

### Background and Objectives

Recently, heated tobacco products (HTPs) have become increasingly popular in Japan. According to the 2020 National Health and Nutrition Survey conducted by the Ministry of Health, Labour and Welfare (MHLW) [1], 27.2% of male and 25.2% of female habitual smokers in Japan used HTPs. In a survey from Japan [2], 47.5% of smokers perceived that HTPs were less harmful to their health than cigarettes. In another survey [3], the most common reason for using HTPs was the belief that HTPs are less harmful than cigarettes. These surveys indicated that many smokers might start using HTPs for their health. However, the long-term effects of HTPs on health have not been clarified, although there are some data indicating that exposure to harmful substances is lower with HTPs than cigarettes [4]. In addition, the belief that HTPs might help quit smoking was also reported as a reason for using HTPs [3]. However, no studies have demonstrated that HTPs help people stop smoking cigarettes, according to a Cochrane report in 2022 [5]. Thus, smokers may use HTPs based on uncertain information that HTPs are beneficial for their health.

A recent survey in Japan reported that smokers with cancer, chronic obstructive pulmonary disease (COPD) or cardiovascular disease (CVD) were more likely to use HTPs over cigarettes than those without the disease [6]. This may be attributable to widespread recognition among smokers that cigarette smoking can cause and worsen cancer [7,8,9], COPD [10,11] or CVD [12,13] and that smokers with these diseases are advised to stop smoking.

Cigarette smoking is also related to the risk of chronic inflammatory diseases (CIDs) such as rheumatoid arthritis (RA) [14,15,16,17], psoriasis [18,19] or atopic dermatitis (AD) [20]. However, it is not known whether smokers with CIDs tend to use HTPs due to their diseases.

The objective of this study was to assess the prevalence of HTP use among smokers with RA, one of the representative CIDs, and to further assess the reasons for HTP use and perceptions about the harmfulness of HTPs among smokers with RA. It is considered that this survey will serve as a foundation for providing better education on smoking cessation to smokers with RA. To the best of our knowledge, this is the first survey to assess the actual use of HTPs among smokers with RA.

## 2. Materials and Methods

In this study, we conducted a web questionnaire survey in Japan between December 2020 and January 2021 using a research company panel (Macromill Carenet (Tokyo, Japan)), one of the largest commercial web panels in Japan with more than 8.6 million panelists. This study was reviewed and approved by the Research Ethics Committee of Nagoya University (no. 2020-0338).

### 2.1. Patient and Public Involvement

Patients or the public were not involved in the design, conduct, reporting or dissemination plans of our study.

### 2.2. Participants

**Figure 1:**
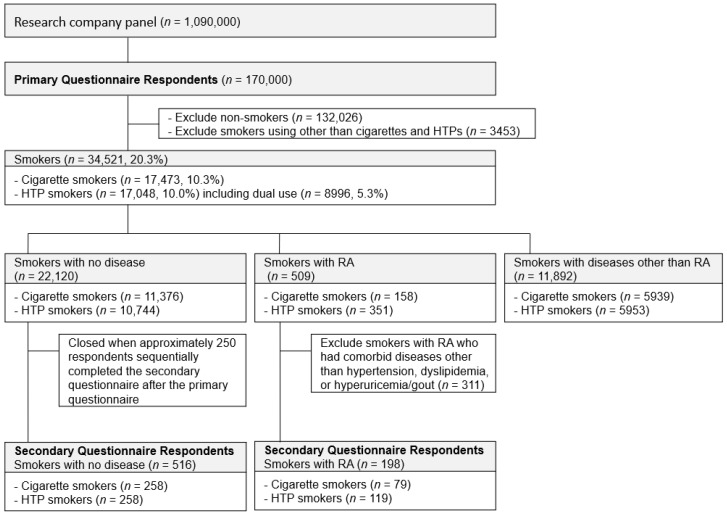
Study participants. A total of 170,000 respondents from 1,090,000 panelists completed the primary questionnaire. A total of 516 smokers with no disease and 198 smokers with RA sequentially completed the secondary questionnaire. HTP: heated tobacco product, RA: rheumatoid arthritis.

Participants were recruited on a simple random basis from panelists aged 20 years or older in a large survey panel managed by Macromill Carenet, an internet research agency in Japan. The respondents were required to provide web-based consent to participate in this study. We prepared two questionnaires (Table S1), one for a primary questionnaire regarding smoking and disease status to assess the prevalence of HTP use in smokers with RA, and the other for a secondary questionnaire regarding the reasons for HTP use and perceptions about the harmfulness of HTPs. We planned to administer the primary questionnaire to 170,000 respondents and sequentially administer the secondary questionnaire to those who answered to the primary questionnaire by any of the following: (a) cigarette smokers with RA, (b) HTP smokers with RA, (c) cigarette smokers with no disease and (d) HTP smokers with no disease, with a cap of approximately 250 per group (Figure 1). In the secondary questionnaire, smokers with RA who had comorbid diseases other than hypertension, dyslipidemia or hyperuricemia/gout were excluded from the respondents to avoid the impact of these comorbidities on the interpretation of results.

### 2.3. Variables

#### 2.3.1. Current Smoking Status (Cigarettes, HTPs, Dual Use of Cigarettes and HTPs)

The primary questionnaire collected self-reported information on current smoking status (non-smoking, smoking with cigarettes, smoking with HTPs, smoking with both cigarettes and HTPs and others). In this study, the following definitions were used to classify respondents: “non-smokers”, those who currently did not smoke; “cigarette smokers”, those who currently used only cigarettes; “HTP smokers”, those who currently used HTPs including smokers with dual use of cigarettes and HTPs. 

#### 2.3.2. Current Disease Status

The primary questionnaire collected self-reported information on the current presence of the following diseases: RA, psoriasis, AD, hypertension, diabetes, dyslipidemia, hyperuricemia/gout, CVD, hepatic disease, kidney disease, COPD, cancer and others. The secondary questionnaire also collected RA duration and activity, and presence or absence of prior hospitalization due to RA. 

#### 2.3.3. General Information

The primary questionnaire collected self-reported information on sex, age, residence (metropolitan area, other area) and equivalent household income (<4.0, 4.0–7.9, 8.0–11.9, ≥12.0 million JPY). The secondary questionnaire also collected education (junior high school, high school, university, technical school, graduate school, other), drinking habits, number of cigarettes smoked per day (including HTPs) and presence of cohabitants.

#### 2.3.4. Details of HTP Use and Perceptions about HTPs

The secondary questionnaire collected the following self-reported information:

- reasons for HTP use;
- perceptions about the negative impact of HTPs on RA;
- consideration of HTP use due to RA;
- perceptions about whether RA is ameliorated by starting HTPs;
- changes in smoking frequency after starting HTPs.

### 2.4. Statistical Analysis

The target sample size of smokers with RA in the primary questionnaire was planned to be 500 respondents; this sample size was considered to provide a sufficient 95% confidence interval (CI) with a margin of errors of 4.4% at the widest point and was expected to yield approximately 100 respondents for each of HTP smokers with RA and cigarette smokers with RA in the secondary questionnaire.

Participant characteristics were tabulated by number and percentage for each item. Variables were analyzed in univariate and multivariate analyses. *p* values < 0.05 were considered statistically significant. The Chi-square test or Fisher’s exact test was conducted for cigarette smokers vs. HTP smokers, and for smokers with no disease vs. smokers with RA. Logistic regression analysis was used to calculate adjusted odds ratios (ORs) with 95% CIs for each disease (e.g., with RA vs. without RA), with HTP use employed as a response variable. Age, sex, equivalent household income and residence were included as explanatory variables for adjustment. Analyses were based on observed cases without imputation of missing data. Analyses were conducted using the IBM SPSS statistics software (v. 28; IBM Corp, Armonk, NY, USA).

## 3. Results

### 3.1. Results of the Primary Questionnaire

#### 3.1.1. The Prevalence of HTP Smokers with RA

The primary questionnaire was sent to 1,090,000 panelists; 170,000 (15.6%) completed the questionnaire (Figure 1). The proportion of smokers among the respondents was 20.3% (cigarette smokers: 10.3%, HTP smokers: 10.0% (HTP only: 4.7%, dual use: 5.3%)). Regarding current disease status, 22,120 smokers responded that they had no current disease and 12,401 responded they had at least one disease, including 509 smokers with RA (Table 1). 

The prevalence of HTP use in all smokers was 49.4%, and that in smokers with RA was 69.0%. The prevalence of HTP use by disease was as follows: 69.3% for COPD, 69.0% for RA, 65.8% for psoriasis, 62.8% for kidney disease, 60.9% for cancer, 59.4% for hepatic disease, 58.9% for AD, 57.7% for hyperuricemia/gout, 54.6% for dyslipidemia, 53.3% for CVD, 50.1% for diabetes, 49.6% for hypertension, 45.8% for other diseases and 48.6% for no current disease.

#### 3.1.2. Analysis of Predictors of HTP Use

The univariate analysis showed that age (<60 years old), sex (male), equivalent household income (JPY ≥ 4 MM) and residence in a metropolitan area were significantly related to HTP use. The presence of RA, AD, psoriasis, dyslipidemia, hyperuricemia/gout, CVD, hepatic disease, kidney disease, COPD and cancer was also significantly related to HTP use. 

**Figure 2:**
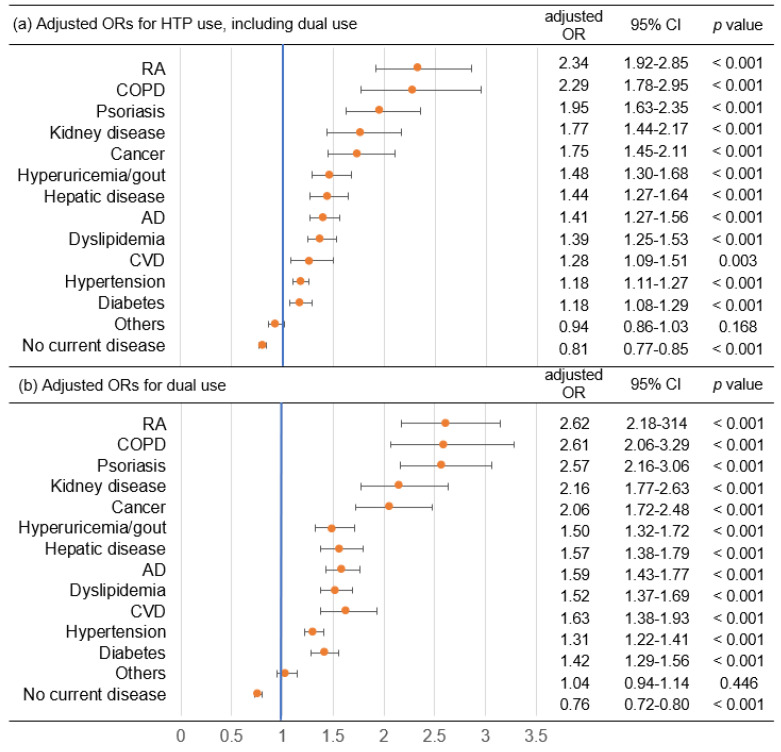
Adjusted ORs of diseases for HTP use with adjustment for age, sex, equivalent household income and residence. (**a**) Adjusted ORs for HTP use including dual use of cigarettes and HTPs. (**b**) Adjusted ORs for dual use of cigarettes and HTPs. HTP: heated tobacco product, OR: odds ratio, CI: confidence interval, RA: rheumatoid arthritis, COPD: chronic obstructive pulmonary disease, AD: atopic dermatitis, CVD: cardiovascular disease.

Logistic regression analysis was conducted to calculate the adjusted ORs and 95% CIs of each disease for HTP use with adjustment for age, sex, equivalent household income and residence (Figure 2). The adjusted OR with 95% CI of RA for HTP use including dual use was 2.34 (1.92, 2.85) (*p* < 0.001) and that of COPD was 2.29 (1.78, 2.95) (*p* < 0.001). The adjusted OR with 95% CI of RA for dual use of cigarettes and HTPs was 2.62 (2.18, 3.14) (*p* < 0.001) and that of COPD was 2.61 (2.06, 3.29) (*p* < 0.001).

### 3.2. Results of the Secondary Questionnaire

#### 3.2.1. Characteristics of Participants who Completed the Secondary Questionnaire

Following the primary questionnaire of 170,000 respondents, 516 smokers with no disease (cigarette smokers: *n* = 258, HTP smokers: *n* = 258) and 198 smokers with RA (cigarette smokers: *n* = 79, HTP smokers: *n* = 119) sequentially completed the secondary questionnaire (Table 2).

The univariate analysis showed that among both smokers with RA and smokers with no disease, the presence of a drinking habit, high education level and the presence of cohabitants were significantly related to HTP use, in addition to age and equivalent household income found in the primary questionnaire. Among smokers with RA, the presence of prior hospitalization due to RA was related to HTP use, but not disease activity or disease duration (Table 3).

#### 3.2.2. Main Results from the Secondary Questionnaire

Responses regarding the best reason for HTP use are shown in Table 4(a). The proportion of HTP smokers with RA who answered “Less harmful to health” was 24.4%, which was not significantly different from that of HTP smokers with no disease (27.1%) (*p* = 0.571). 

Among smokers with RA, the opinions about the negative impacts of cigarettes and HTPs on RA are shown in Table 4(b). The proportion of respondents who answered “Cigarettes are more harmful than HTPs” was numerically higher among HTP smokers with RA (55.5%) than among cigarette smokers with RA (45.6%), but the difference was not statistically significant (*p* = 0.173).

Responses about whether respondents had ever considered using HTP due to RA are shown in Table 4(c). The proportion of respondents who answered “Yes” was significantly higher among HTP smokers with RA (43.7%) than among cigarette smokers with RA (25.3%) (*p* = 0.008). 

Perceptions about whether switching to HTPs or dual use of HTPs and cigarettes relieves RA are shown in Table 4(d). The proportion of respondents who answered “Yes” was significantly higher among HTP smokers with RA (42.0%) than among cigarette smokers with RA (21.5%) (*p* = 0.008). 

Responses about actual or anticipated changes in the frequency of smoking (either cigarettes or HTPs) after starting HTPs are shown in Table 4(e). An increase in frequency after starting the use of HTPs was reported by 33.0% of smokers with RA. By contrast, an anticipated increase among participants who had not yet used HTPs was reported by 6.3% of cigarette smokers with RA (*p* < 0.001).

## 4. Discussion

The results of the primary questionnaire in this study revealed that smokers with RA were more likely to use HTPs than those without RA, and the tendency was higher compared with other diseases. The secondary questionnaire revealed that 43.7% of HTP smokers with RA had considered using HTPs due to RA, and 42.0% of them felt that switching to HTPs or dual use of HTPs relieved their RA symptoms. This indicated that smokers with RA tend to start using HTPs due to their RA, despite the lack of scientific evidences to support that HTPs are safer alternatives to cigarettes for RA. 

The data obtained in this study are consistent with previous reports. The proportion of smokers among the panelists who completed the primary questionnaire (20.3%) in this study is similar to the proportion of 16.7% in the 2020 National Health and Nutrition Survey conducted by the MHLW1. The proportions of HTP use including dual use of cigarettes and dual use (10.0%, 5.3%) in this study are comparable to the values of 9.0% and 6.1% in the previous study published by Odani et al. in 2020 [21]. As for the prevalence of RA, the proportion of the total respondents with RA was 0.85% (1446/170,000), which is consistent with the previously reported prevalence of RA in Japan (0.5–1.0%) [22]. These results suggest that the panel used in this study accurately reflected the actual situation in Japan.

In a previous survey regarding the prevalence of HTP use by disease by Nakama et al. [13], diabetes, CVD, COPD and cancer were correlated with HTP use. Similar results were observed in this study, and it was newly found that RA and other CIDs, such as psoriasis and AD, were also corelated with HTP use. This study showed smokers with RA were as likely as smokers with COPD. This finding is of interest given that COPD patients are likely to perceive the direct negative impact of smoking on subjective symptoms of COPD, such as dyspnea, cough or sputum, whereas RA patients are considered less likely to perceive the direct impact of smoking on RA symptoms and possibly less motivated to quit smoking.

Among smokers with RA, prior hospitalization due to RA was related to HTP use; however, the disease activity was not related, contrary to our expectations that more severe RA patients would be more likely to use HTPs. This is possibly because disease activity was assessed by respondents themselves and was not certified by physicians. 

In some previous reports [13,21], sex, drinking habit and use of e-cigarettes were reported to correlate with HTP use. The factors related to HTP use identified in this study, low age, high income, high education level and presence of cohabitants, may be attributable to (i) HTPs are relatively new, (ii) they produce less smoke and odor, (iii) their use is more complex and (iv) a larger up-front cost is required. 

The proportion of respondents who used HTPs because they were “Less harmful to health than cigarettes” was unexpectedly similar between HTP smokers with RA and HTP smokers with no disease. Assuming that smoking is generally accepted as harmful because it is associated with lung cancer [23], this questionnaire might have led respondents to primally think about this association. On the other hand, the proportion of respondents who answered “Cigarettes are more harmful than HTPs for RA” was more than half of HTP smokers with RA, suggesting that this might be an important motivation to use HTPs. In addition, the proportion of respondents who answered “considered using HTPs because of RA” was significantly higher among HTP smokers with RA (43.7%) than among cigarette smokers with RA (25.3%), which directly showed that RA motivated smokers with RA to use HTPs.

Surprisingly, 42.0% of HTP smokers with RA stated that they felt “that the use of HTPs relieves RA”. This perception about the positive impact of HTPs on RA is not scientifically supported since disease activity was not assessed by physicians in this study. However, it is important information showing what HTP smokers with RA actually feel. Furthermore, 33.3% of HTP smokers with RA stated that “Smoking frequency was increased after starting HTPs,” while 6.3% of cigarette smokers with RA anticipated that this frequency would increase if they started using HTPs. Thus, in terms of smoking frequency, there was a gap between what smokers with RA predicted before HTP use and the actual change after HTP use. Initiating HTP use on the basis of uncertain information that HTPs are less harmful than cigarettes is considered to be a major problem, given the risk of increased smoking frequency.

## 5. Limitations

This study has some potential limitations. The data, including disease information, were obtained from an internet survey completed by respondents themselves and were not certified by physicians. However, to enhance the reliability of the results, questionnaire items such as those on disease activity were accompanied by additional information (e.g., definitions of “mild,” “moderate,” and “severe” for disease activity) to increase the accuracy of respondents’ replies. Analyses based on observed cases without imputation of missing data could be a potential limitation. We conducted two questionnaires by sequential approach, which may have caused selection bias. Another limitation is that for the secondary questionnaire, the sample size of cigarette smokers with RA was small, which might have resulted in an underpowered analysis. In addition, as this study did not include minors under 20 years of age, prohibited from smoking in Japan, there was a possibility of selection bias. Some questions regarding past experiences and conditions may be associated with recall bias.

## 6. Conclusions

In conclusion, this study revealed that smokers with RA were likely to use HTPs. In addition, nearly half of the HTP smokers with RA had considered using HTPs due to their RA and felt that, stating HTP relieved their RA symptoms. These results indicated that smokers with RA tend to start using HTPs due to their RA despite the lack of scientific evidences to support that HTPs are safer alternatives to cigarettes for RA. Since the adverse effects of HTPs on RA are not fully understood [24], additional research should address this question. Furthermore, HTPs may prevent smokers with RA from quitting smoking completely, which can adversely affect their long-term disease control. Finally, it is important to inform smokers with RA that it is unknown how HTP use may affect their disease and to provide them with proper smoking cessation guidance.

## Figures and Tables

**Table 1:** Characteristics of participants who completed the primary questionnaire.

	Total Smokers	Cigarette Smokers	HTP Smokers	*p* Value
	*n* (%)	*n* (%)	*n* (%)	
Number	34,521 (100.0)	17,473 (50.6)	17,048 (49.4)	
Sex				
Male	25,440 (73.7)	12,499 (45.2)	12,941 (54.8)	<0.001
Female	9081 (26.3)	4974 (54.8)	4107 (45.2)	
Age (years)				
<60	27,637 (80.0)	13,051 (47.2)	14,586 (52.8)	<0.001
≥60	6884 (19.9)	4422 (64.2)	2462 (35.8)	
Residence				
Metropolitan area	13,223 (38.3)	6537 (49.4)	6686 (50.6)	<0.001
Other area	21,298 (61.7)	10,936 (51.3)	10,362 (48.7)	
Equivalent household income (JPY)				
<4 MM	9017 (26.1)	5282 (58.6)	3735 (41.4)	<0.001
≥4 MM	19,438 (56.3)	8927 (45.9)	10,511 (54.1)	
Did not know/Unwilling to answer	6066 (17.6)	3264 (53.8)	2802 (46.2)	
Current disease				
Hypertension	4898 (14.2)	2469 (50.4)	2429 (49.6)	0.754
Diabetes	2324 (6.7)	1160 (49.9)	1164 (50.1)	0.484
Dyslipidemia	1882 (5.5)	854 (45.4)	1028 (54.6)	<0.001
AD	1765 (5.1)	725 (41.1)	1040 (58.9)	<0.001
Hyperuricemia/Gout	1089 (3.2)	461 (42.3)	628 (57.7)	<0.001
Hepatic disease	1080 (3.1)	439 (40.6)	641 (59.4)	<0.001
CVD	655 (1.9)	306 (46.7)	349 (53.3)	0.044
Psoriasis	571 (1.7)	195 (34.2)	376 (65.8)	<0.001
Cancer	517 (1.5)	202 (39.1)	315 (60.9)	<0.001
RA	509 (1.5)	158 (31.0)	351 (69.0)	<0.001
Kidney disease	435 (1.3)	162 (37.2)	273 (62.8)	<0.001
COPD	309 (0.9)	95 (30.7)	214 (69.3)	<0.001
Others	2627 (7.6)	1423 (54.2)	1204 (45.8)	<0.001
No current disease	22,120 (64.1)	11,376 (51.4)	10,744 (48.6)	<0.001

Cigarette smokers: smokers who currently used only cigarettes, HTP smokers: smokers who currently used HTPs including smokers with dual use of cigarettes and HTPs, Http: heated tobacco product, JPY: Japanese Yen, MM: million, AD: atopic dermatitis, CVD: cardiovascular disease, RA: rheumatoid arthritis, COPD: chronic obstructive pulmonary disease.

**Table 2:** Characteristics of participants who completed the secondary questionnaire.

	Smokers with No Disease		*p* Value	Smokers with RA		*p* Value
	Cigarette Smokers (*n* = 258)	HTP Smokers (*n* = 258)		Cigarette Smokers (*n* = 79)	HTP Smokers (*n* = 119)	
	*n* (%)	*n* (%)		*n* (%)	*n* (%)	
Sex						
Male	221 (85.7)	228 (88.4)	0.359	41 (51.9)	74 (62.2)	0.151
Female	37 (14.3)	30 (11.6)		38 (48.1)	45 (37.8)	
Age (years)						
<60	203 (78.7)	236 (91.5)	<0.001	47 (59.5)	92 (77.4)	0.007
≥60	55 (21.3)	22 (8.5)		32 (40.5)	27 (22.7)	
Residence						
Metropolitan area	104 (40.3)	98 (38.0)	0.588	23 (29.1)	49 (41.2)	0.084
Other areas	154 (59.7)	160 (62.0)		56 (70.9)	70 (58.8)	
Equivalent household income (JPY)						
<4 MM	80 (31.0)	53 (20.5)	<0.001	24 (30.4)	23 (19.3)	0.032
≥4 MM	126 (48.8)	169 (65.5)		39 (49.3)	79 (66.4)	
Did not know/Unwilling to answer	52 (20.2)	36 (14.0)		16 (20.3)	17 (14.3)	
Drinking habit						
Absent	68 (26.4)	40 (15.5)	0.002	31 (39.2)	29 (24.4)	0.026
Present	190 (73.6)	218 (84.5)		48 (60.8)	90 (75.7)	
Number of cigarettes/HTPs smoked per day						
≤10	96 (37.2)	94 (36.4)	0.855	28 (35.4)	43 (36.1)	0.921
>11	162 (62.8)	164 (63.6)		51 (64.6)	76 (63.8)	
Education level						
Junior high school/High school/Other	106 (41.1)	76 (29.5)	0.005	44 (55.7)	38 (31.9)	0.003
University/Technical school/Graduate school	136 (52.7)	165 (64.0)		34 (43.0)	71 (59.7)	
Unwilling to answer	3 (1.2)	2 (0.8)		0 (0.0)	5 (4.2)	
Presence of cohabitants						
Absent	62 (24.0)	43 (16.7)	0.038	21 (26.6)	12 (10.1)	0.002
Present	196 (76.0)	215 (83.3)		58 (73.4)	107 (89.9)	

Cigarette smokers: smokers who currently used only cigarettes, HTP smokers: smokers who currently used HTPs including smokers with dual use of cigarettes and HTPs. HTP: heated tobacco product, RA: rheumatoid arthritis, JPY: Japanese Yen, MM: million.

**Table 3:** Disease information of smokers with RA who completed the secondary questionnaire.

	Smokers with RA		*p* Value
	Cigarette Smokers (*n* = 79)	HTP Smokers (*n* = 119)	
Disease duration	*n* (%)	*n* (%)	
<5 years	37 (46.8)	40 (33.6)	0.091
≥5 years	41 (51.9)	73 (61.3)	
Did not know	1 (1.3)	6 (5.0)	
Disease activity			
Mild	45 (57.0)	70 (58.8)	0.795
Moderate to Most severe	34 (43.0)	49 (41.2)	
Prior hospitalization due to RA			
Yes	16 (20.3)	40 (33.6)	0.041
No	63 (79.7)	79 (66.4)	

Cigarette smokers: smokers who currently used only cigarettes, HTP smokers: smokers who currently used HTPs including smokers with dual use of cigarettes and HTPs, HTP: heated tobacco product, RA: rheumatoid arthritis.

**Table 4:** Summary of the results of the secondary questionnaire.

**(a) Reasons for HTP Use**	**HTP Smokers with No Disease** **(*n* = 258)**	**HTP Smokers with RA** **(*n* = 119)**	***p*** **Value**
What is the best reason to use HTPs?	*n* (%)	*n* (%)	
Less harmful to health	70 (27.1)	29 (24.4)	0.571
Less smoke and odor	136 (52.7)	63 (52.9)	
Taste	17 (6.6)	19 (16.0)	
Appearance/Fashion	8 (3.1)	4 (3.4)	
More smoking area	23 (8.9)	4 (3.4)	
Other	4 (1.6)	0 (0)	
**(b) Perceptions Regarding the Negative Impact of HTPs on RA**	**Smokers with RA**		***p*** **Value**
	**Cigarette Smokers** **(*n* = 79)**	**HTP Smokers** **(*n* = 119)**	
Do you think cigarettes and HTPs differ in their negative impact on RA?	*n* (%)	*n* (%)	
Yes—Cigarettes are more harmful	36 (45.6)	66 (55.5)	0.173
Yes—HTPs are more harmful	4 (5.1)	17 (14.3)	
No	39 (49.4)	36 (30.3)	
**(c) Consideration of HTP Use Due to RA**	**Smokers with RA**		***p*** **Value**
	**Cigarette Smokers** **(*n* = 79)**	**HTP Smokers** **(*n* = 119)**	
Have you ever considered using HTPs due to RA?	*n* (%)	*n* (%)	
Yes	20 (25.3)	52 (43.7)	0.008
No	59 (74.7)	67 (56.3)	
**(d) Perceptions about whether RA Is Ameliorated by Starting HTPs**	**Smokers with RA**		***p*** **Value**
	**Cigarette Smokers** **(*n* = 79)**	**HTP Smokers** (*n* = 100) *	
Do you feel that the use of HTPs relieves RA? (If you are not using HTPs, answer based on what you think might occur.)	*n* (%)	*n* (%)	
Yes	17 (21.5)	42 (42.0)	0.008
No	62 (78.5)	58 (58.0)	
**(e) Changes in Smoking Frequency after Starting HTPs**	**Smokers with RA**		***p*** **Value**
	**Cigarette Smokers** **(*n* = 79)**	**HTP Smokers** (*n* = 100) *	
After starting HTPs, did your smoking frequency change?/Do you think your smoking frequency will change once you start using HTPs?	*n* (%)	*n* (%)	
Yes—Increased/Will increase	5 (6.3)	33 (33.0)	< 0.001
Yes—Decreased/Will decrease	27 (34.2)	33 (33.0)	
No	47 (59.5)	34 (34.0)	

(a) Reasons for HTP use, (b) perceptions on the negative impact of HTPs on RA, (c) consideration of HTP use due to RA, (d) perceptions about whether RA is ameliorated by starting HTPs and (e) changes in smoking frequency after starting HTPs. * Respondents without experience smoking cigarettes are not included. HTP: heated tobacco product, RA: rheumatoid arthritis.

## Data Availability

Data are available on reasonable request. Data may be made available by the authors upon review of reasonable request.

## Supplementary Materials

The following are available online at https://www.mdpi.com/article/10.3390/ijerph191912465/s1, Table S1. The primary questionnaire and the secondary questionnaire.
