# Supplementary material for: A Survey on the Actual Use of and Reasons for Heated Tobacco Products in Patients with Rheumatoid Arthritis
Source: Int J Environ Res Public Health. 2022 Sep 30;19(19):12465. doi: 10.3390/ijerph191912465 (PMC9564473; doi:10.3390/ijerph191912465)
Supplement: Supplementary file 1 [file ijerph-19-12465-s001.zip › ijerph-1925987-SI.pdf]

Table S1. The primary questionnaire and the secondary questionnaire

**Primary questionnaire**	
Questions	Responses
1. Sex	Male, Female
2. Age	__ years
3. What area do you live in?	Hokkaido, Tohoku, Kanto, Chubu, Kinki, Chugoku, Shikoku, Kyushu
4. Equivalent household income (million Japanese Yen)	-1.9, 2.0-3.9, 4.0-7.9, 8.0-11.9, 12.0-, Do not know, Unwilling to answer
5. Do you currently smoke?	Do not smoke, Only cigarettes, Only HTPs, HTPs and cigarettes
6. Which of the following diseases are you currently suffering from?<br>*Must be diagnosed by a medical institution.<br>*Multiple answers possible	Hypertension, Diabetes, Dyslipidemia, Hyperuricemia/gout, Cardiovascular disease, Hepatic disease, Kidney disease, Rheumatoid arthritis, Chronic obstructive pulmonary disease, Cancer, Atopic dermatitis, Psoriasis, Others, No disease
**Secondary questionnaire**	
Questions	Responses
1. How often do you drink?	None, ≤ 1 per month, 2-4 per month, 2~3 per week, ≥ 4 times a week
2. How many cigarettes do you smoke on average per day?<br>*Please answer the combined number of cigarettes and HTPs.	-10, 11-20, 21-30, 31-
3. Please tell us your academic history.	Junior high school, High school, University or Technical school, Graduate school, Other, Unwilling to answer
4. Who are your family living together?	Living alone, Parents, Brothers and sisters, Parents, brothers and sisters, Spouse, Children, Spouse and children, Parents and spouse, Parents, spouse and children, Other
5. How long you have been diagnosed with rheumatoid arthritis?	-4.9, 5-9.9, 10-19.9, 20-, Do not know
6. Which of the following conditions best	Mild, Moderate, Severe, Most severe

describes to your current disease status of rheumatoid arthritis?<br>*Class 1 to 4 in the following definition correspond to mild, moderate, severe, and most severe in this order.<br>I - Complete ability to carry out all the usual duties without handicaps<br>II - Adequate for normal activities despite handicap of discomfort or limited motion of one of the joints<br>III - Limited to little or none of the duties of usual occupation or self-care<br>IV - Incapacitated, largely or wholly bed-ridden or confined to a wheelchair with little or no self-care<br>https://www.mhlw.go.jp/new-info/kobetu/kenkou/ryumachi/dl/jouhou01-11-0003.pdf	
7. Have you ever been hospitalized due to rheumatoid arthritis?	Yes, No
8. How did you get started with HTPs?	Switch from cigarettes or add to cigarettes, From the beginning
9. Please select one option that best describes the reason for HTPs use.	Less harmful to the health, Less smoke and odor, Taste, Appearance/Fashion, More smoking area, Other
10. Do you think cigarettes and HTPs differ in their negative impact on your rheumatoid arthritis?	Yes - Cigarettes are more harmful, Yes - HTPs are more harmful, No
11. Have you ever considered using HTPs due to your rheumatoid arthritis?	Yes, No
12. Do you feel that the use of HTPs relieves your disease? (If you are not using HTPs, answer based on what you think might occur)	Yes, No
13. After starting HTPs, did your smoking frequency change? / Do you think smoking frequency will change once you start using HTP?	Yes - Increased, Yes - Decreased, No / Yes - Will increase, Yes - Will decrease, No
